# Economic hardship and mental health following a national trauma and amid ongoing conflict: a longitudinal cohort study

**DOI:** 10.1007/s00127-026-03180-7

**Published:** 2026-08-28

**Authors:** Dana Katsoty, Yaakov Greenwald, Yuval Neria, Ariel Knafo-Noam, Ido Lurie

**Affiliations:** 1 Psychology Department, The Hebrew University of Jerusalem, Jerusalem, Israel; 2 Department of Psychiatry, Columbia University Irving Medical Center, New York, NY USA; 3 New York State Psychiatric Institute, Columbia University Irving Medical Center, New York, USA; 4 Shalvata Mental Health Center, Hod Hasharon, Israel; 5 Grey School of Medicine, Tel Aviv University, Tel Aviv, Israel

**Keywords:** Economic hardship, Mental health, War exposure, Depression and anxiety, Post-traumatic stress disorder, Longitudinal research

## Abstract

**Purpose:**

Exposure to war and terrorism increases the risk of mental health problems and often leads to economic hardship, which can further heighten psychological distress. This longitudinal study examined the extent of economic hardship experienced during the Israel–Hamas war following the October 7th terrorist attack, the severity of mental health symptoms over the first year of the war, and the associations and temporal directionality between economic hardship and mental health.

**Methods:**

A representative sample of Jewish Israeli adults (*N* = 1,035) completed surveys at four time points: two weeks, and two, six, and 12 months after the attack. Participants reported economic hardship events (e.g., job loss, reduced income, unpaid leave, increased expenses, business losses) and symptoms of depression, anxiety, and posttraumatic stress disorder (PTSD).

**Results:**

Psychological symptoms remained elevated throughout the year, declining from two weeks to two and six months after the attack, but rising again by 12 months. Economic hardship was prevalent and consistently associated with worse mental health outcomes. Longitudinal analyses showed a dynamic pattern: in the early stage of the war, poorer mental health predicted subsequent economic hardship, whereas later in the year, economic hardship predicted increases in depression, anxiety, and PTSD symptoms.

**Conclusion:**

Psychological distress and economic hardship mutually influence one another across different stages of prolonged mass trauma. Integrating economic assistance with psychological support may help reduce long-term mental health impacts.

**Supplementary Information:**

The online version contains supplementary material available at https://doi.org/10.1007/s00127-026-03180-7.

## Introduction

Prolonged, nationwide exposure to war may carry profound consequences across various domains of life, including both mental health and economic well-being [1, 2]. The economic effects of war exposure are often referred to as the indirect effects of war [3], and operate both on the macro level (e.g., reduced economic growth, declining tax revenues, and conflict-related budget cuts [4]) and the micro level (diminished wealth, income loss, asset depletion, and reduced consumption [5, 6]). Even the threat of violence, in the absence of direct exposure, has been linked to adverse individual economic outcomes [5, 7], which may in turn heighten vulnerability to psychological distress. This distinction is particularly important given the wide variability in war exposure across populations, with many individuals experiencing primarily threat, without direct attacks [6]. The current research longitudinally investigates the toll that war has on mental health and economic hardship, and the dynamic relationships between these two domains over time.

Economic hardships are consistently associated with adverse mental health outcomes [8–10]. Lower socioeconomic status (SES), including lower income, limited education, unemployment, and economic instability, is a well-established risk factor for depression, anxiety, and posttraumatic stress disorder (PTSD), and is associated with higher prevalence and greater severity of PTSD symptoms [11–15]. For instance, unemployment is associated with a 1.5-fold increase in PTSD risk, and economic instability significantly predicts PTSD symptom severity [16]. Broader contextual factors, such as neighborhood disadvantage and income inequality, further elevate risk: for example, state-level income inequality has been linked to higher rates of PTSD among trauma-exposed adults [17]. Notably, these associations persist after accounting for trauma exposure and social support, underscoring SES as an independent risk factor [11, 12, 14].

A central issue in understanding the link between economic hardship and mental health is the directionality of the association. Two competing hypotheses are social causation (low SES increases mental health risk) and social drift or selection (mental health problems lead to SES declines [18, 19]). Longitudinal studies support both directions of influence and potential reciprocal effects. Both subjective and objective SES measures show bidirectional association with mental health outcomes (e.g., depression, anxiety) over a 20-year period, with stronger effects for subjective SES [20]. Additionally, prior exposure to traumatic events and resulting PTSD increase the risk of subsequent material hardship, while earlier material hardship raises the likelihood of developing PTSD, supporting both the social causation and social selection hypotheses [21]. This bidirectional relationship has been observed across diverse populations and appears largely independent of gender [13, 21].

In the context of war exposure, the economic hardship-mental health association may manifest in distinct ways. Vulnerability varies with baseline SES, access to resources and pre-existing health conditions [22]. War-related economic hardship may create a “double burden”, where the trauma of conflict combined with economic strain jointly intensify mental health symptoms and limit access to treatment and recovery.

While growing research has demonstrated the detrimental mental health implications of the October 7th attacks and the Israel-Hamas war [23–26], research on the economic impact of the war and its association with mental health outcomes remains limited. One study reported elevated depression, anxiety, and PTSD symptoms among individuals experiencing economic hardship in the first three months of the war, using a binary hardship measure (experiencing vs. not experiencing [27]). However, no research to date has examined the association between the severity of economic hardship and mental health over longer periods of time, nor the potential bidirectional effects between economic hardship and mental health outcomes. To address these gaps, we aimed to assess the following domains longitudinally: (a) the population-level extent of economic hardship during the Israel-Hamas war and the severity of depression, anxiety, and PTSD symptoms; (b) the associations between the severity of economic hardship and mental health outcomes; and (c) the longitudinal relationships and the direction of effects between economic hardship and mental health outcomes.

## Method

### Participants and procedure

Data for this study were collected as part of a large longitudinal project conducted following the October 7th, 2023 attack and the ensuing Israel-Hamas war [28]. A representative sample of Jewish adults stratified by gender, age, and religiosity was recruited through the survey company Panel4All. Recruitment of a representative Arab-Israeli sample was not feasible due to insufficient sample size within the survey registry. Participants provided informed consent at each wave. The study protocol and procedures were approved by the Institutional Review Board of the Hebrew University. Data were collected at four waves following the October 7th attack: two weeks (on average, mid-late October 2023, T1); two months (mid-November to early December 2023, T2); 6 months (mid-late April 2024, T3); one year post-attack (early-mid October 2024, T4). Of the initial T1 sample (*N* = 1,124), 89 participants were excluded due to survey non-completion or failure to pass all attention checks, yielding a final analytic T1 sample of 1,035, who were recontacted for subsequent waves. After exclusion of participants who failed attention checks at later waves, sample sizes were 801 (43 excluded) at T2, 670 (21 excluded) at T3, and 660 (21 excluded) at T4. Attrition analyses were conducted and reported in the Results section.

### Measures

#### Sociodemographic variables

Participants reported age, sex, education, religion, and religious group (i.e., secular, traditional, religious, ultra-orthodox) at T1.

#### Depression and anxiety symptoms

These symptoms were measured at all waves using the Patient Health Questionnaire-4 (PHQ-4[29]). Participants reported the extent to which they experienced the feelings described in each item over the past two weeks on a 4-point Likert scale (0 = *not at all*, 3 = *almost every day*). The questionnaire includes two items for depression and two items for anxiety. Cronbach’s alpha indicated good internal consistency for both scales across waves (*α* = 0.82 − 0.86 for anxiety, *α* = 0.72 − 0.77 for depression). Cutoff scores were used for descriptive purposes and to assess overall symptom severity. A score of 3 or higher for each of the depression and anxiety scales was used to indicate clinically significant symptoms [29].

#### PTSD symptoms

PTSD symptoms were measured at T3 and T4 (six months and one year after the attack) using the Posttraumatic Stress Disorder Checklist (PCL-5) [30], which includes the 20 symptoms listed in the Diagnostic and Statistical Manual of Mental Disorders [18]. Participants rated the extent to which the feelings, thoughts, or behaviors described in each item bothered them over the past month, specifically in relation to the Israel-Hamas war, on a 5-point Likert scale (1 = *not at all*, 5 = *extremely*). The total score was computed as the sum of all items. Internal consistency was excellent (*α* = 0.96 at both waves). The clinical cutoff for the PCL-5 score was 33[31].

#### Exposure to the events of the war

At T1, using items adapted from prior research [32, 33], participants answered yes (1)/no (0) questions about their direct and indirect exposure, including personal injuries and injuries or deaths of close others, rocket damage to their home or nearby areas, military service of themselves, and displacement. The total level of exposure was computed as the sum of all items (possible range: 0–10).

#### Economic hardship during the war

At T2-T4, participants were asked to indicate whether they or their partner experienced workplace loss or furlough (unpaid leave), and whether they experienced a decrease in income due to fewer work hours, an increase in expenses, or loss of income from a business. The sum of reported items indicated the level of economic hardship. In additional analyses, this variable was clustered into three groups based on severity level, defined by the number of negative events (with participants experiencing up to 4 events): none = 0 events, moderate = 1–2 events, and high = 3–4 events.

#### General income

At T1, participants reported their general household income level relative to the mean in Israel (from 1 = A lot below average, to 5 = A lot above average).

### Statistical analysis

All analyses were conducted in R (R Core Team, 2022). Analyses were conducted in three main steps to examine associations between economic hardship and mental health outcomes across time. First, logistic regressions were used to predict binary probable diagnoses for depression, anxiety, and PTSD, based on whether participants’ symptom scores exceeded established clinical cutoffs. Economic hardship served as the predictor, and the regressions included covariates (general income, level of exposure to the events of the war, age, gender) and odds ratios with 95% confidence intervals were reported.

Second, multilevel linear mixed-effects models were used to examine the association between economic hardship and mental health outcomes (depression, anxiety, PTSD, continuous) over time, accounting for repeated measures within participants via random intercepts. Economic hardship was modeled as an ordered factor to test for a linear effect across severity levels (none, moderate, high), and time was modeled as an ordered factor as well. When a significant overall effect was detected, post hoc pairwise comparisons using the Benjamini–Hochberg correction were conducted to compare symptom severity across hardship levels. Models were estimated using restricted maximum likelihood (REML).

Finally, longitudinal path analyses were conducted to examine temporal associations and direction of effects between economic hardship and each of the mental health outcomes. Each model included autoregressive (within-construct) paths, cross-lagged (between-construct) paths, and within-time covariances, with model fit evaluated using standard indices. Models with CFI values > 0.90 were considered to have an acceptable fit and models with a CFI > 0.95 were considered to have a good fit; RMSEA and SRMR values < 0.08 indicated an acceptable fit, and < 0.05 indicated a good fit [34].

## Results

### Sample characteristics

Table 1 presents the sample characteristics, along with mental health outcomes and economic hardship across waves. At T1 (*N* = 1,035), the mean age of participants was 43.93 years (SD = 16.10). Almost all participants (1020, 98.8%) identified as Jewish. Gender distribution was about equal for men and women (534, 51.6% women). The mean number of years of education was 14.82 (SD = 2.8). Most participants identified as secular (438, 42.4%).

### Attrition analyses

We began by comparing participants who participated in 1, 2, 3 or 4 waves of the study to test for differences in study variables (depression, anxiety, PTSD, economic hardship) and additional variables (income, age, gender, war exposure level) using a series of univariate ANOVA analyses (and a chi-square test for gender), accounting for multiple comparisons using Benjamini–Hochberg FDR correction. Main effects were found for age and PTSD at T4. For PTSD at T4 and age, we conducted post hoc Tukey tests, again applying FDR correction. For age, participants who completed more waves tended to be older. The 3-wave group was significantly older than the 1-wave group (M difference = 7.81 years, *p* <.001). The 4-wave group was older than all other groups, with significant differences relative to the 1-wave (M difference = 12.72 years, *p* <.001), 2-wave (M difference = 8.78 years, *p* <.001), and 3-wave groups (M difference = 4.85 years, *p* =.001). For PTSD, the 4-wave group had lower scores than the 3-wave group (M difference = − 5.7, *p* =.007).

### Mental health

At T1, over half of participants exceeded clinical cutoffs for depression (55.8%) and anxiety (61.1%). Rates decreased by T2, where over one-third of participants exceeded clinical cutoffs for depression (35.8%) and anxiety (37.5%). There was another decrease by T3 (26.6% and 26.0% for depression and anxiety, respectively), followed by a rise by T4 (27.6% and 31.1% depression and anxiety, respectively). PTSD, assessed only at T3 and T4, increased from 16.9% to 21.4% (See Table 1 for full results).

### Economic hardship

Economic hardship was widespread. Most participants reported one or two of the possible events at each time point (65.9%, 49.6%, 52.6% at T2, T3, T4, respectively; see SM Table 1 for full results).

### Descriptive correlations

Correlations between main study variables – depression, anxiety, PTSD, economic hardship, and general income – are presented in Table 1 in the Supplementary Material (SM Table 1). All variables measured longitudinally were correlated across measurements: e.g., depression at T1 was strongly associated with depression at T2-T4; economic hardship at T2 was strongly related to economic hardship at T3 and T4. All mental health measures (depression, anxiety, PTSD) were strongly correlated within and between time points. Level of exposure to the events of the war was positively, yet generally weakly, associated with most mental health outcomes across time, and with economic hardship at T2 and T4. General income was negatively, yet weakly, associated with economic hardship, suggesting that higher income was associated with lower risk of experiencing adverse economic events. General income was negatively associated with some of the subsequent mental health outcomes (e.g., anxiety and PTSD at T2 and T3).

**Table 1:** Demographic characteristics of participants and mental health and economic hardship across timepoints (*N* = 1,035)

Items	M (SD)		*n* (%)	
Age (years) (T1)	43.93 (16.1)			
Education (years) (T1)	14.82 (2.8)			
Income level (T1)	2.50 (1.25)			
Gender (T1)				
Women			534 (51.6)	
Men			498 (48.1)	
Non-binary/other			3 (0.3)	
Religiosity (T1)				
Secular			438 (42.4)	
Traditional			372 (36.0)	
Religious			132 (12.8)	
Ultra-orthodox			80 (7.8)	
Other			10 (1.0)	

Mental health outcomes	* n* (%) above clinical cutoff			
	T1	T2	T3	T4
Depression	576 (55.8)	286 (35.8)	178 (26.6)	182 (27.5)
Anxiety	631 (61.1)	300 (37.5)	174 (26.0)	205 (31.1)
PTSD		-	113 (16.9)	141 (21.4)

Economic hardship	* n* (%) per group			
	T1	T2	T3	T4
None (0 events)	-	241 (30.1)	306 (45.7)	270 (40.9)
Moderate (1–2 events)	-	528 (65.9)	332 (49.6)	347 (52.6)
High (3–4 events)	-	32 (4.0)	32 (4.8)	43 (6.5)

### Concurrent associations: logistic regressions

Economic hardship was positively associated with all mental health outcomes at all waves (Table 1). To address the role of economic hardship beyond gender and age, as well as general income and level of war exposure, we conducted logistic regression analyses. Of note, since only 3 participants − 0.3% - identified as other/nonbinary, they were dropped from the analysis, and gender was addressed as dichotomous. These analyses indicated that greater economic hardship was consistently associated with increased odds of meeting criteria for a probable diagnosis of depression, anxiety, and PTSD, controlling for all covariates (Table 2). Across all waves, higher levels of economic hardship were significantly associated with a greater likelihood of scoring above the clinical cutoff for each mental health outcome, with odds ratios ranging from 1.25 to 1.48.

**Table 2:** Concurrent logistic regressions predicting a probable diagnosis

Model	Economic Hardship OR (95% CI)	General income OR (95% CI)	Exposure level OR (95% CI)	Age OR (95% CI)	Gender OR (95% CI)
Depression T2	1.48 [1.23, 1.79]***	1.03 [0.91, 1.16]	0.93 [0.81, 1.07]	0.99 [0.98, 1.00]	2.21 [1.63, 3.01]***
Depression T3	1.43 [1.17, 1.74]**	0.97 [0.83, 1.12]	1.16 [0.98, 1.37]	0.99 [0.98, 1.00]	2.20 [1.52, 3.20]***
Depression T4	1.25 [1.04, 1.50]*	0.89 [0.76, 1.03]	1.16 [0.99, 1.36]	1.00 [0.99, 1.01]	1.73 [1.20, 2.49]**
Anxiety T2	1.43 [1.18, 1.73]**	1.02 [0.90, 1.16]	1.04 [0.90, 1.20]	0.99 [0.98, 1.00]	3.31 [2.42, 4.53]***
Anxiety T3	1.47 [1.20, 1.80]***	0.90 [0.77, 1.05]	1.22 [1.03, 1.44]*	0.99 [0.98, 1.00]	2.95 [2.02, 4.36]***
Anxiety T4	1.37 [1.14, 1.64]**	0.89 [0.76, 1.03]	1.12 [0.96, 1.32]	1.00 [0.98, 1.01]	2.47 [1.73, 3.54]***
PTSD T3	1.42 [1.14, 1.76]**	0.99 [0.83, 1.18]	1.15 [0.95, 1.39]	0.97 [0.96, 0.99]***	1.90 [1.23, 2.97]**
PTSD T4	1.35 [1.11, 1.64]**	0.84 [0.71, 0.99]	1.03 [0.86, 1.23]	0.97 [0.96, 0.99]***	1.91 [1.27, 2.88]**

Note. OR = odds ratio; CI = confidence interval. *p* values adjusted using Benjamini-Hochberg correction (***p <.001, **p <.01, *p <.05)

### Mixed-effects models

Three mixed-effects models examined the association between mental health and economic hardship across time (see Table 3 for full results of all models). Regarding change in mental health outcomes, significant longitudinal effects emerged for all measures. Depression and anxiety symptoms decreased over time (*b* = − 1.04, 95% CI [− 1.14, − 0.95], and *b* = − 1.09, 95% CI [− 1.19, − 0.99], respectively). However, significant quadratic effects were also found for both depression and anxiety (*b* = 0.57, 95% CI [0.48, 0.66], and *b* = 0.72, 95% CI [0.62, 0.81], respectively), indicating a curvilinear trajectory, with symptoms initially declining and then rising again at the latest wave. Similarly, PTSD symptoms increased over time (*b* = 0.77, 95% CI [0.09, 1.46]). Of note, a quadratic effect could not be examined as PTSD was measured only at T3 and T4.

**Fig. 1:**
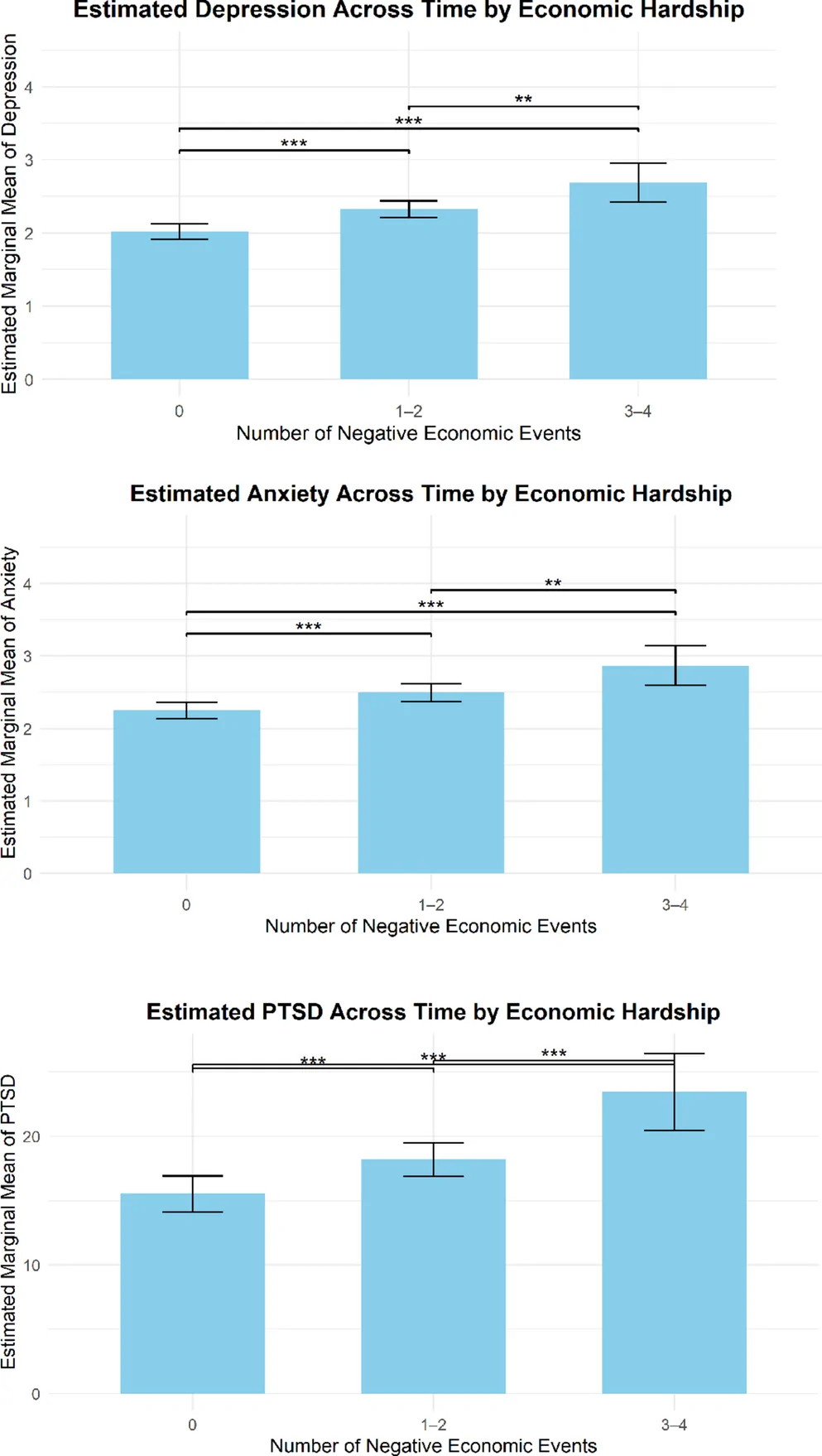
Estimated marginal means for depression, anxiety, and PTSD symptoms across levels of economic hardship with pairwise comparisons

Regarding the association between mental health outcomes and economic hardship, a significant linear effect of economic hardship was found for all three outcomes, with higher levels of hardship predicting greater symptom severity (depression: *b* = 0.47, 95% CI [0.28, 0.67]; anxiety: *b* = 0.44, 95% CI [0.24, 0.64]; PTSD: *b* = 5.61, 95% CI [3.36, 7.85]). Pairwise comparisons of the estimated marginal means indicated a clear dose–response pattern, with significant differences between all levels of economic hardship for each outcome (all *p* <.01, after Benjamini–Hochberg correction). Figure 1 presents the estimated marginal means of depression, anxiety, and PTSD symptoms across number of economic hardship events with pairwise comparisons.

**Table 3:** Results of linear mixed-effects models for depression, anxiety, and PTSD by economic hardship

Predictors	Depression	Anxiety	PTSD
	Estimates	Estimates	Estimates
(Intercept)	2.35 (2.23–2.47)	2.54 (2.41–2.67)	19.06 (17.72–20.39)
Economic hardship [linear]	0.47 (0.28–0.67)	0.44 (0.24–0.64)	5.61 (3.36–7.85)
Economic hardship [quadratic]	0.02 (−0.10–0.15)	0.05 (−0.08–0.18)	1.06 (−0.37–2.50)
Time [linear]	−1.04 (−1.14 – −0.95)	−1.09 (−1.19 – −0.99)	0.77 (0.09–1.46)
Time [quadratic]	0.57 (0.48–0.66)	0.72 (0.62–0.81)	
**Random Effects**			
σ^2^	1.28	1.33	71.75
τ_00_	1.63	2.05	182.37
ICC	0.56	0.61	0.72
N	1034	1035	763
Observations	3163	3166	1328
Marginal R^2^/Conditional R^2^	0.098/0.603	0.107/0.649	0.017/0.722
Deviance	11343.977	11621.786	10710.015
AIC	11384.736	11662.141	10718.726
log-Likelihood	−5684.368	−5823.070	−5353.363

Note. The table presents fixed-effect estimates with 95% CIs and random-effect parameters from multilevel models. σ² = within-person variance; τ₀₀ = between-person variance; ICC = intraclass correlation. Marginal R² = variance explained by fixed effects; Conditional R² = variance explained by fixed and random effects. A quadratic effect could not be assessed for PTSD as it was measured twice

### Longitudinal path analyses models

**Fig. 2:**
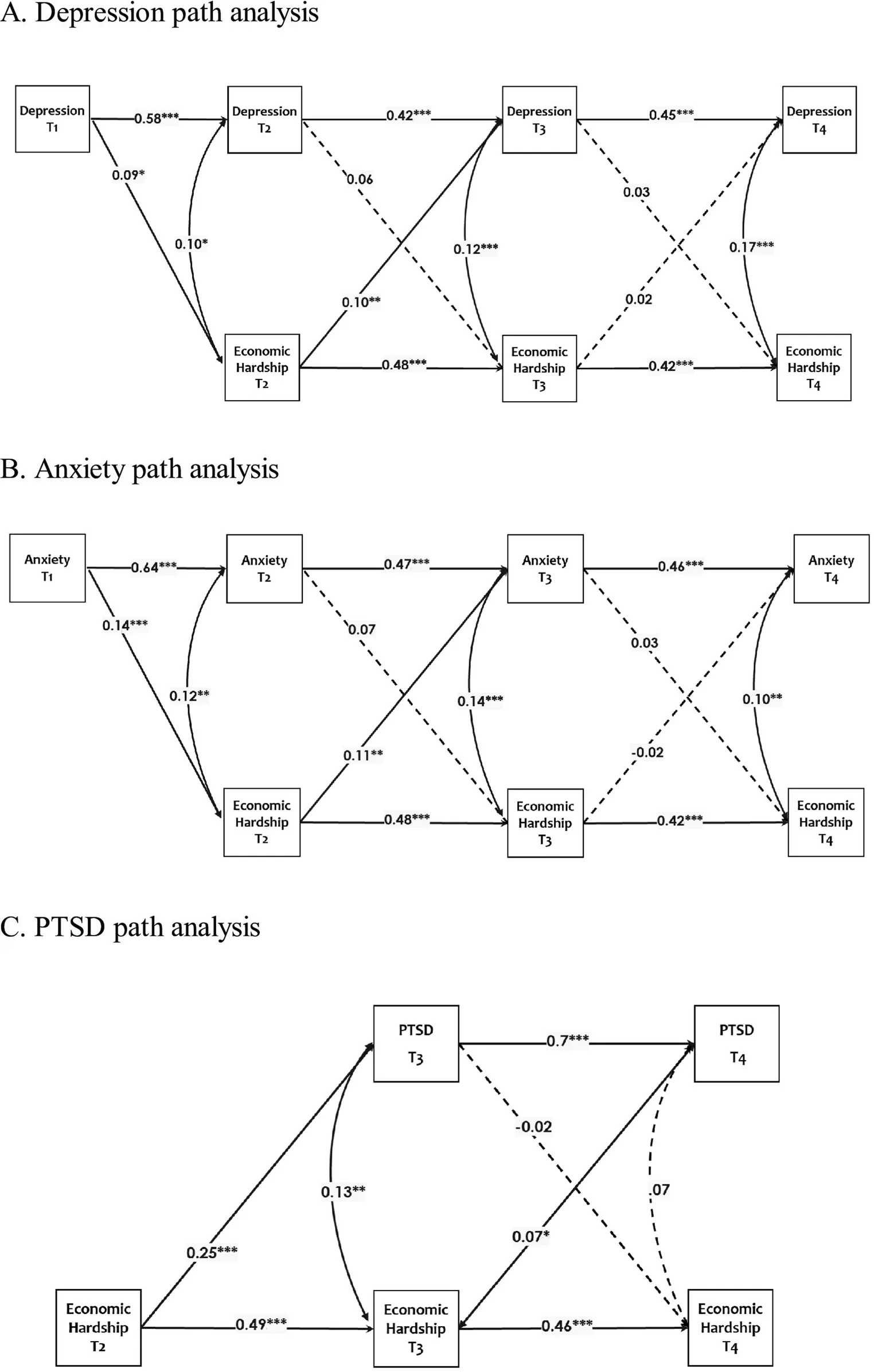
Results of the three path analysis models for economic hardship and depression, anxiety and PTSD, respectively. For clarity of presentation, non-consecutive autoregressive paths were omitted from the figure and are reported in the Supplementary Material (SM Table 2), which includes full model results. Path coefficients are standardized. Black lines represent significant effects (***p <.001, **p <.01, *p <.05), dashed lines represent nonsignificant effects

Finally, to further examine the associations between economic hardship and mental health across time and test the direction of effects, we conducted three separate path analysis models linking economic hardship to depression, anxiety, and PTSD. Each model included autoregressive paths for each variable across timepoints, cross lagged paths between variables in consecutive timepoints, and covariances between variables within each timepoint. Standardized results of the models are presented in Fig. 2, excluding non-consecutive autoregressive paths for clarity of presentation. Full results of all models are presented in Table 2 in the Supplementary Material (SM Table 2).

All models had excellent fit to the data. Model fit indices were as follows: Depression: *χ²*(4) = 8.09, *p* =.088, CFI = 0.997, RMSEA = 0.032, SRMR = 0.014; Anxiety: *χ²*(4) = 8.477, *p* =.076, CFI = 0.997, RMSEA = 0.033, SRMR = 0.013; PTSD: *χ²*(1) = 3.72, *p* =.054, CFI = 0.997, RMSEA = 0.058, SRMR = 0.011.

In all models, all autoregressive paths and covariances were significant, indicating that the constructs were stable across time and that there were associations between economic hardship and mental health outcomes within each wave. The depression and anxiety models showed a similar pattern: depression and anxiety at T1 predicted economic hardship at T2, and economic hardship at T2 predicted depression and anxiety at T3. No other significant cross lagged paths were found. The model examining PTSD (which, as noted, was not measured at T1 and T2) showed that economic hardship at T2 predicted PTSD at T3, and economic hardship at T3 predicted PTSD at T4.

## Discussion

This longitudinal study provides a comprehensive examination of mental health, economic hardship, and their associations during the first year of the Israel–Hamas war, drawing on data from four assessment waves. The findings extend a growing body of literature documenting the wide-ranging effects of war on mental health [35, 36] and its indirect economic consequences [5, 6].

Across all mental health outcomes, symptom severity was substantial, consistent with other research highlighting the psychological toll of the ongoing war [27, 37]. However, despite the continuous nature of trauma exposure throughout the study period, symptoms declined during the first six months of the war and then increased by the one-year mark. One possible explanation relates to the dual nature of trauma exposure in this context: an acute traumatic component (the October 7th attack) alongside a continuous trauma component (the ensuing war). Within this framework, the initially elevated symptoms may reflect expected acute stress reactions during a period of peak national shock, widespread fear, uncertainty, and extensive media exposure following the attack, rather than enduring psychopathology. Accordingly, although exposure to ongoing trauma persisted, the initial symptom decline may represent a recovery pattern similar to those observed following other large-scale acute trauma events, such as the September 11th attacks [38]. As immediate threat perceptions stabilized and aspects of daily routines gradually resumed, distress levels may have attenuated over time.

Another possible explanation involves the strong national solidarity and widespread social support that characterized the Israeli public response during the early months of the war. Social cohesion and collective support are well-established protective factors following collective trauma and may have temporarily buffered psychological distress [39, 40]. Additionally, some individuals may initially function in a heightened “crisis mode,” mobilizing coping resources during the period closer to the acute trauma exposure [41]. Symptoms may have increased again by the one-year mark because the protective effects of collective coping and heightened resilience may diminish under conditions of prolonged conflict. Sustained exposure to threat—including rocket attacks, military operations, and persistent uncertainty—may have gradually depleted coping resources and psychological resilience, consistent with cumulative stress models proposing that prolonged stress can overwhelm adaptive capacities [42]. Moreover, the initial surge of social solidarity may have weakened over time as the conflict extended beyond the acute crisis phase, potentially intensifying feelings of helplessness and demoralization.

Finally, the economic consequences of the war, reflected in the substantial levels of hardship documented in our study, likely became increasingly cumulative over time. As financial reserves diminished and employment disruptions persisted, economic stress may have compounded the direct psychological burden of the conflict. Consistent with this interpretation, our findings showed that economic hardship predicted subsequent mental health outcomes, suggesting that accumulating hardship may have contributed to the later increase in symptoms.

It is also important to note that the findings regarding the increase in symptoms from 6 months to one year into the war partly diverge from prior research on mass-trauma exposure, which shows a steady post-event decline in symptoms [43], and from some longitudinal findings related to the current war [44, 45]. However, they align with recent evidence indicating symptom escalation between 6 months and one-year post-attack [46]. Of note, attrition analyses further indicated that participants who remained in the study across all waves reported lower PTSD levels at the latest measurement compared to those who participated at only three waves, suggesting that overall PTSD levels in the broader population may be underestimated in the current work. Additionally, younger participants (who also demonstrated higher distress levels) were less likely to remain in the study over time, which may have contributed to an underestimation of symptom severity.

Economic hardship reported by participants was substantial. Across all waves, most participants were in the moderate economic hardship group (1–2 events), reflecting the notable economic impact of the war. General income, reported shortly after the outbreak of the war, was found to have small but statistically significant negative correlations with economic hardship across all waves, suggesting that individuals with lower pre-war income were more likely to experience economic hardship during the war. This pattern is consistent with the “Matthew effect” [47], whereby pre-existing socioeconomic disadvantage amplifies vulnerability during crises.

Associations between mental health and economic variables (general income and economic hardship) were complex and time dependent. General income level was not associated with depression and anxiety in the early phases of the war, but small negative associations emerged at six and twelve months. In contrast, the associations between war-related economic hardship and mental health outcomes were robust and consistent: the number of negative economic events was significantly correlated with all mental health outcomes across waves. Examining the direction of effects revealed that depression and anxiety soon after the war predicted economic hardship two months later, and that economic hardship at two months predicted depression, anxiety, and PTSD six months into the war (economic hardship 6 months into the war also predicted PTSD one year into the war).

Although economic hardship was not measured at the earliest wave, limiting our ability to assess very early economic effects on mental health, our findings align with prior evidence documenting bidirectional associations between mental health and economic hardship [20, 21] even during large-scale external crises beyond the individual’s control. This pattern may reflect a potential vicious cycle. Specifically, the finding that initial psychological distress predicted later economic hardship may suggest that early mental health symptoms compromised individuals’ capacity to effectively manage daily demands, make adaptive decisions, or maintain stable functioning, thereby increasing their vulnerability to economic strain. In turn, subsequent economic hardship predicted later psychological distress, further eroding mental health over time. Such reciprocal reinforcement may be particularly pronounced during prolonged national crisis, in which both stress and economic instability are sustained.

These reciprocal effects are particularly noteworthy given the unique context of nationwide exposure, where economic hardships might have been expected to be less indicative of personal characteristics (e.g., psychological vulnerability or prior functioning) and more reflective of shared structural conditions affecting individuals across the socioeconomic and psychological spectrum. For example, during the early stages of the war, economic activity across the country came to a near-complete halt [48], and such widespread disruptions could potentially reduce the influence of individual-level factors on exposure to economic hardship. Yet, even given the nation-level stressor affecting the entire population, there was evidence of effects from mental health to economic hardship and vice versa. This may indicate that people experiencing mental health difficulties in the first place are more likely to be affected by economic conditions during the war.

Importantly, economic hardship showed notably stronger associations with mental health than did war exposure. These associations remained significant after controlling for the level of war exposure, underscoring the unique and robust link between the two domains. Of note, war exposure was measured cumulatively to reflect the reality that many individuals experienced layered or compounded exposure to multiple war-related stressors across the course of the war. However, this measure included exposure to events that likely differed substantially in nature and personal impact (e.g., loss of a loved one versus military service or displacement), which may have reduced the specificity of the cumulative exposure score and attenuated the exposure to war effects. For example, many individuals were exposed primarily to ongoing rocket attacks (Katsoty et al., 2024), which still allowed substantial parts of the population to maintain some degree of daily routine, particularly during the later stages of the war.

In contrast, economic hardship may have had a more persistent impact on daily functioning, perceived control, and future security. Additionally, it is possible that part of the psychological impact of war exposure operated indirectly through secondary economic consequences. Individuals exposed to displacement, reserve duty, rocket attacks, or other war-related disruptions may have subsequently experienced employment instability, income loss, or increased financial strain, which in turn may have contributed to psychological distress over time. It is also important to note that many individuals experienced high levels of fear and uncertainty regardless of their objective exposure level. Although examining nuanced forms of exposure was beyond the primary scope of the current study, future research would benefit from addressing the complexity and nuance of different exposure types and their associations with mental health outcomes.

Several strengths of the study should be mentioned. First, it employed objective and comprehensive measures of economic hardship, encompassing multiple domains, including increased expenses, income loss, and job loss experienced by either the participant or their partner. This is particularly important given that subjective perceptions of financial strain may be influenced by mood or mental health status, potentially introducing rater bias. In addition, the longitudinal design enabled a detailed examination of both mental health and economic hardship throughout the first year of the war, providing valuable insights into the direction of effects, and showing evidence for bidirectional effects at different times of exposure.

It is also essential to address the study’s limitations. First, the study relied on self-reports of income and psychiatric symptom severity, without independent verification of income, quantification of economic impact, or the use of clinical diagnoses, which are generally considered the preferred standard for assessing psychological difficulties [49]. Additionally, the sample consisted of Jewish participants, without representation of the large Arab minority in Israel, which is characterized by a lower socioeconomic status [50]. Previous studies have reported elevated psychological distress within this population, particularly during times of war [51], highlighting the importance of examining these issues among Arab citizens of Israel as well.

Furthermore, because economic hardship was not assessed at T1, any prospective effects it may have had on later mental health could not be examined. Likewise, because PTSD was not measured at T1 and T2, we were unable to test a more comprehensive model of directional effects, comparable to those estimated for depression and anxiety. It is therefore possible that the absence of effects from PTSD to economic hardship reflects a measurement limitation, and that similar to anxiety and depression, PTSD may have also shown prospective effects on economic hardship had we assessed it at the earlier stages.

## Conclusions and policy implications

Results indicated that individuals with lower socioeconomic status experienced more economic hardship during the war. Mental health symptoms soon after the October 7th attack predicted later economic hardship, and economic hardship, in turn, predicted increases in subsequent mental health symptoms - suggesting a potential escalation cycle in which psychological distress and financial strain mutually reinforce one another, albeit at different timepoints. This bidirectional pattern highlights the need for public mental health frameworks that address psychological and economic needs simultaneously. National emergency and rehabilitation programs should therefore integrate psychological interventions with socioeconomic support and improved access to care. Such integrated approaches may prevent a “double burden” in which financial distress intensifies the mental health consequences of war exposure, help alleviate individual suffering, and reduce future societal costs.

## Supplementary Information

Below is the link to the electronic supplementary material.

Supplementary Material 1

## Data Availability

Study materials and data are available upon reasonable request.
