# Supplementary material for: Economic hardship and mental health following a national trauma and amid ongoing conflict: a longitudinal cohort study
Source: Soc Psychiatry Psychiatr Epidemiol. 2026 Aug 28;61(12):2695–705. doi: 10.1007/s00127-026-03180-7 (PMC13727910; doi:10.1007/s00127-026-03180-7)
Supplement: Supplementary file 1 [file 127_2026_3180_MOESM1_ESM.docx]

**SM Table 1**

*Means, standard deviations, and correlations between study variables*

| Variable | *M* | *SD* | 1 | 2 | 3 | 4 | 5 | 6 | 7 | 8 | 9 | 10 | 11 | 12 | 13 | 14 |
| --- | --- | --- | --- | --- | --- | --- | --- | --- | --- | --- | --- | --- | --- | --- | --- | --- |
|  |  |  |  |  |  |  |  |  |  |  |  |  |  |  |  |  |
| 1. Exposure T1 | 0.88 | 1.08 |  |  |  |  |  |  |  |  |  |  |  |  |  |  |
|  |  |  |  |  |  |  |  |  |  |  |  |  |  |  |  |  |
| 2. Income T1 | 2.51 | 1.25 | .02 |  |  |  |  |  |  |  |  |  |  |  |  |  |
|  |  |  |  |  |  |  |  |  |  |  |  |  |  |  |  |  |
| 3. Econ. hardship T2 | 0.94 | 0.81 | .17** | -.10** |  |  |  |  |  |  |  |  |  |  |  |  |
|  |  |  |  |  |  |  |  |  |  |  |  |  |  |  |  |  |
| 4. Econ. Hardship T3 | 0.77 | 0.88 | .07 | -.09* | .47** |  |  |  |  |  |  |  |  |  |  |  |
|  |  |  |  |  |  |  |  |  |  |  |  |  |  |  |  |  |
| 5. Econ. Hardship T4 | 0.86 | 0.92 | .08* | -.13** | .43** | .52** |  |  |  |  |  |  |  |  |  |  |
|  |  |  |  |  |  |  |  |  |  |  |  |  |  |  |  |  |
| 6. Depression T1 | 3.00 | 1.87 | .10** | .00 | .08* | .14** | .10** |  |  |  |  |  |  |  |  |  |
|  |  |  |  |  |  |  |  |  |  |  |  |  |  |  |  |  |
| 7. Depression T2 | 2.23 | 1.67 | .05 | -.04 | .18** | .13** | .09* | .57** |  |  |  |  |  |  |  |  |
|  |  |  |  |  |  |  |  |  |  |  |  |  |  |  |  |  |
| 8. Depression T3 | 1.65 | 1.58 | .09* | -.07 | .18** | .23** | .16** | .52** | .59** |  |  |  |  |  |  |  |
|  |  |  |  |  |  |  |  |  |  |  |  |  |  |  |  |  |
| 9. Depression T4 | 1.82 | 1.65 | .12** | -.10** | .18** | .16** | .18** | .50** | .59** | .67** |  |  |  |  |  |  |
|  |  |  |  |  |  |  |  |  |  |  |  |  |  |  |  |  |
| 10. Anxiety T1 | 3.36 | 1.99 | .13** | -.06 | .13** | .16** | .12** | .78** | .56** | .48** | .47** |  |  |  |  |  |
|  |  |  |  |  |  |  |  |  |  |  |  |  |  |  |  |  |
| 11. Anxiety T2 | 2.27 | 1.83 | .07 | -.04 | .17** | .15** | .08 | .54** | .81** | .56** | .56** | .64** |  |  |  |  |
|  |  |  |  |  |  |  |  |  |  |  |  |  |  |  |  |  |
| 12. Anxiety T3 | 1.79 | 1.70 | .12** | -.12** | .22** | .25** | .17** | .50** | .56** | .79** | .60** | .57** | .64** |  |  |  |
|  |  |  |  |  |  |  |  |  |  |  |  |  |  |  |  |  |
| 13. Anxiety T4 | 2.01 | 1.76 | .10* | -.14** | .20** | .15** | .18** | .44** | .52** | .59** | .79** | .53** | .61** | .68** |  |  |
|  |  |  |  |  |  |  |  |  |  |  |  |  |  |  |  |  |
| 14. PTSD T3 | 16.20 | 15.64 | .11** | -.10** | .24** | .23** | .14** | .40** | .48** | .65** | .56** | .44** | .50** | .67** | .57** |  |
|  |  |  |  |  |  |  |  |  |  |  |  |  |  |  |  |  |
| 15. PTSD T4 | 18.12 | 16.62 | .08 | -.18** | .23** | .22** | .20** | .40** | .49** | .59** | .63** | .45** | .53** | .65** | .66** | .72** |

*Note*. M and SD are used to represent mean and standard deviation, respectively. (****p* < .001, ***p* < .01, **p* < .05).

**SM Table 2**

*SM Table 2A.* Path Analysis Model Results: Depression

| **Dependent Variable / Predictor** | **B** | **Β** (**standardized**) | ***p*** |
| --- | --- | --- | --- |
| **Depression** **T4** on: |  |  |  |
| Depression T3 | 0.47 | 0.44 | <0.001 |
| Depression T2 | 0.25 | 0.26 | <0.001 |
| Depression T1 | 0.10 | 0.12 | 0.001 |
| Economic hardship T3 | 0.04 | 0.02 | 0.538 |
| **Economic hardship T4** on: |  |  |  |
| Economic hardship T3 | 0.44 | 0.42 | <0.001 |
| Economic hardship T2 | 0.27 | 0.23 | <0.001 |
| Depression T3 | 0.02 | 0.03 | 0.396 |
| **Depression** **T3** on: |  |  |  |
| Depression T2 | 0.40 | 0.42 | <0.001 |
| Depression T1 | 0.21 | 0.25 | <0.001 |
| Economic hardship T2 | **0.19** | **0.10** | **0.004** |
| **Economic hardship T3** on: |  |  |  |
| Economic hardship T2 | 0.53 | 0.48 | <0.001 |
| Depression T2 | 0.03 | 0.06 | 0.106 |
| **Depression** **T2** on: |  |  |  |
| Depression T1 | 0.52 | 0.58 | <0.001 |
| **Economic hardship T2** on: |  |  |  |
| Depression T1 | **0.04** | **0.09** | **0.014** |

***Note***. Significant cross-lagged paths are presented in bold.

*SM Table 2B.* Path Analysis Model Results: Anxiety

| **Dependent Variable / Predictor** | **B** | **Β** (**standardized**) | ***p*** |
| --- | --- | --- | --- |
| **Anxiety** **T4** on: |  |  |  |
| Anxiety T3 | 0.47 | 0.46 | <0.001 |
| Anxiety T2 | 0.25 | 0.27 | <0.001 |
| Anxiety T1 | 0.09 | 0.10 | 0.006 |
| Economic hardship T3 | 0.04 | 0.02 | 0.624 |
| **Economic hardship T4** on: |  |  |  |
| Economic hardship T3 | 0.45 | 0.42 | <0.001 |
| Economic hardship T2 | 0.26 | 0.23 | <0.001 |
| Anxiety T3 | 0.02 | 0.03 | 0.423 |
| **Anxiety** **T3** on: |  |  |  |
| Anxiety T2 | 0.44 | 0.47 | <0.001 |
| Anxiety T1 | 0.21 | 0.24 | <0.001 |
| Economic hardship T2 | **0.22** | **0.11** | **0.001** |
| **Economic hardship T3** on: |  |  |  |
| Economic hardship T2 | 0.52 | 0.48 | <0.001 |
| Anxiety T2 | 0.03 | 0.07 | 0.055 |
| **Anxiety** **T2** on: |  |  |  |
| Anxiety T1 | 0.60 | 0.64 | <0.001 |
| **Economic hardship T2** on: |  |  |  |
| Anxiety T1 | **0.06** | **0.14** | **<0.001** |

***Note***. Significant cross-lagged paths are presented in bold.

*SM Table 2C.* Path Analysis Model Results: PTSD

| **Dependent Variable / Predictor** | **B** | **Β** (**standardized**) | ***p*** |
| --- | --- | --- | --- |
| **PTSD** **T4** on: |  |  |  |
| PTSD T3 | 0.72 | 0.70 | <0.001 |
| Economic hardship T3 | **0.23** | **0.07** | **0.047** |
| **Economic hardship T4** on: |  |  |  |
| Economic hardship T3 | 0.48 | 0.46 | <0.001 |
| Economic hardship T2 | 0.26 | 0.22 | <0.001 |
| PTSD T3 | -0.00 | -0.02 | 0.516 |
| **PTSD** **T3** on: |  |  |  |
| Economic hardship T2 | **4.82** | **0.25** | **0.001** |
| **Economic hardship T3** on: |  |  |  |
| Economic hardship T2 | 0.52 | 0.48 | <0.001 |

***Note***. Significant cross-lagged paths are presented in bold.
